# Critical care capacity in Haiti: A nationwide cross-sectional survey

**DOI:** 10.1371/journal.pone.0218141

**Published:** 2019-06-13

**Authors:** Lia I. Losonczy, Sean L. Barnes, Shiping Liu, Sarah R. Williams, Michael T. McCurdy, Vivienne Lemos, Jerry Chandler, L. Nathalie Colas, Marc E. Augustin, Alfred Papali, for the Research and Education consortium for Acute Care in Haiti (REACH) Study Group

**Affiliations:** 1 Division of Pulmonary & Critical Care Medicine, University of Maryland School of Medicine, Baltimore, Maryland, United States of America; 2 Department of Emergency Medicine, George Washington University, Washington, District of Columbia, United States of America; 3 Department of Decision, Operations & Information Technologies, Robert H. Smith School of Business, University of Maryland, College Park, Maryland, United States of America; 4 Department of Mathematics, University of Maryland, College Park, Maryland, United States of America; 5 Taddle Creek Family Health Team, Toronto, Ontario, Canada; 6 Protection Civile Haiti, Port-au-Prince, Haiti; 7 Department of Internal Medicine, St. Luke Hospital, Port-au-Prince, Haiti; 8 Division of Pulmonary & Critical Care Medicine, Atrium Health, Charlotte, North Carolina, United States of America; 9 Division of Pulmonary & Critical Care Medicine, University of North Carolina School of Medicine, Chapel Hill, North Carolina, United States of America; Medical University Graz, AUSTRIA

## Abstract

**Objective:**

Critical illness affects health systems globally, but low- and middle-income countries (LMICs) bear a disproportionate burden. Due to a paucity of data, the capacity to care for critically ill patients in LMICs is largely unknown. Haiti has the lowest health indices in the Western Hemisphere. In this study, we report results of the first known nationwide survey of critical care capacity in Haiti.

**Design:**

Nationwide, cross-sectional survey of Haitian hospitals in 2017–2018.

**Setting:**

Haiti.

**Subjects:**

All Haitian health facilities with at least six hospital beds.

**Interventions:**

Electronic- and paper-based survey.

**Results:**

Of 51 health facilities identified, 39 (76.5%) from all ten Haitian administrative departments completed the survey, reporting 124 reported ICU beds nationally. Of facilities without an ICU, 20 (83.3%) care for critically ill patients in the emergency department. There is capacity to ventilate 62 patients nationally within ICUs and six patients outside of the ICU. One-third of facilities with ICUs report formal critical care training for their physicians. Only five facilities met criteria for a Level 1 ICU as defined by the World Federation of Societies of Intensive and Critical Care Medicine. Self-identified barriers to providing more effective critical care services include lack of physical space for critically ill patients, lack of equipment, and few formally trained physicians and nurses.

**Conclusions:**

Despite a high demand for critical care services in Haiti, current capacity remains insufficient to meet need. A significant amount of critical care in Haiti is provided outside of the ICU, highlighting the important overlap between emergency and critical care medicine in LMICs. Many ICUs in Haiti lack basic components for critical care delivery. Streamlining critical care services through protocol development, education, and training may improve important clinical outcomes.

## Introduction

Critical illness affects health systems across the world, but low- and middle-income countries (LMICs) bear a disproportionate burden [1]. Due to a paucity of data, however, the capacity to care for critically ill patients in LMIC settings is largely unknown. In a recent systematic review, only 15 of 36 low-income countries had any published data regarding their ICU capacity [1]. Only two low-income countries, Nepal and Uganda, had national critical care capacity statistics [1].

The World Health Organization (WHO) advocates for well-defined systems of acute care for the critically ill and injured as an integral part of resilient national health care systems [2]. However, in order to successfully develop such systems in diverse LMIC settings, a thorough characterization and quantification of regional and national acute care capacity, human and material resources, and barriers to capacity growth is essential.

Haiti is among the poorest countries in the world and has the lowest health indices in the Western Hemisphere [3,4]. Recent devastating natural disasters (e.g., hurricane, earthquake) and epidemics (e.g., cholera, chikungunya, zika) have severely strained its healthcare system and demonstrate the critical importance of establishing a robust, integrated acute care system. Recent studies have elucidated some of Haiti’s emergency and trauma care capacity [5–7], but its capacity to provide care for critically ill patients is largely unknown. In this study, we report results of the first known nationwide survey of critical care capacity in Haiti. Beyond establishing the number of intensive care units (ICUs), ICU beds, and ventilators, our goal was to determine how and where critical care is delivered in Haitian hospitals, particularly when no ICU is available.

## Materials and methods

We conducted a cross-sectional nationwide survey of Haitian hospitals in 2017–2018. The study was approved by the St. Luke Hospital (Port-au-Prince, Haiti) Ethics Committee. Consent was not obtained by survey participants as the data were analyzed anonymously.

### List of hospitals

**Fig 1:**
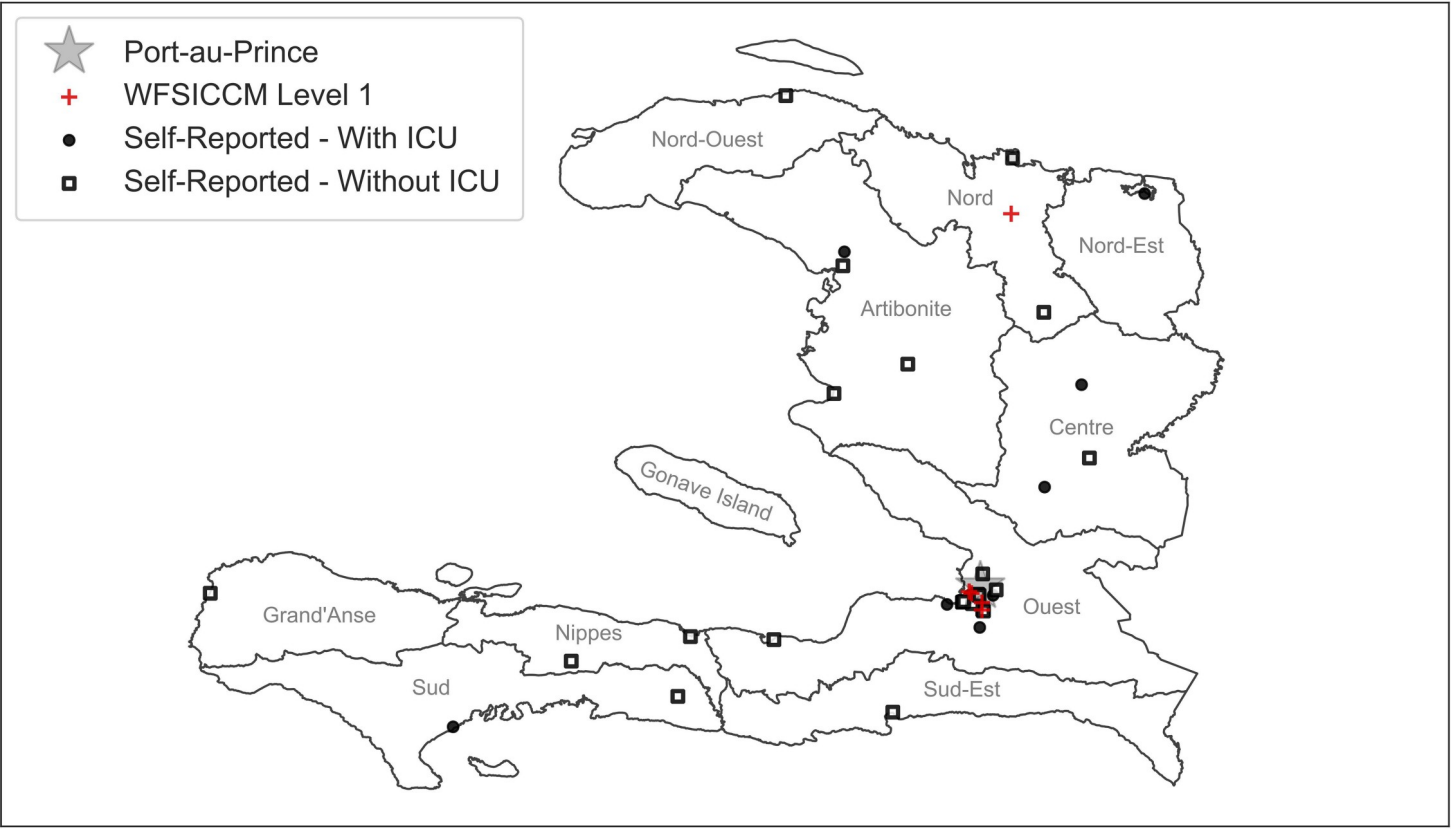
Schematic map of Haiti indicating locations of surveyed facilities.

We first sought to establish an updated list of hospitals and health centers in Haiti. Due to an ever-changing healthcare landscape influenced by economics, politics, natural disasters, and non-governmental organizations (NGOs), many healthcare facilities cease or commence operations in Haiti year-by-year. This fluidity makes an accurate accounting of all functioning hospitals in Haiti very challenging. Starting with the official 2015 Haitian Ministry of Health facility list [8], which distinguishes “hospitals” from “health centers with beds” and “health centers without beds,” we limited our search to facilities with greater than six inpatient beds. We then cross referenced this list with those from the Pan American Health Organization (PAHO) [9] and the United States Embassy in Haiti [10]. Through local health care providers, we subsequently discovered several active hospitals not officially listed by the previously-named organizations, so we widened the search to include internet references [11, 12], and suggestions from Haitian colleagues. We eliminated duplicate facilities, including those colloquially known by different names, based on cross-referencing addresses and by verification of local physicians. We reduced this list to health facilities that we could confirm were still operating by the presence of a valid telephone number, e-mail address, or postal address. Our final list included 48 health facilities throughout the country (Fig 1 and S1 File).

### Creation of survey

A joint task force of US and Haitian physicians adapted a previously-validated survey on critical care capacity for LMICs [13]. After independent review by multiple non-affiliated Haitian physicians and American academicians, the survey was translated from English to French by an independent physician translator, then back-translated to English by a second physician translator. The final survey instrument (S2 and S3 Files) subsequently was piloted in two Haitian hospitals before widespread distribution.

### Collection of data

Surveys were administered electronically via Google Forms in French and English. They were sent to listed medical directors or emergency/critical care physicians at each facility. A Haitian research coordinator conducted the survey in-person at facilities that did not respond electronically, or for which telephone or email contact information was inaccurate. Using multiple choice questions and five-point Likert scales, we collected demographic information on facilities and survey respondents, the presence or absence of emergency departments and ICUs, and the availability of material and human resources to care for critically ill patients. Free text responses were used to identify the most common critical illnesses and the greatest perceived barriers to providing critical care services in their respective institutions.

### Main outcome measures

Primary outcomes were availability and access to equipment, personnel, and therapies relevant for the practice of critical care medicine. Secondary outcomes included identifying barriers to improving care for critically ill patients and adherence to the World Federation of Societies of Intensive and Critical Care Medicine’s (WFSICCM) proposed definition of ICU.

### Data analysis

We summarized categorical variables by frequencies and corresponding percentages for each level. Numerical variables were summarized using a five-number summary (i.e., the minimum, first quartile, median, third quartile, maximum) and the sample mean. In order to compare the most common conditions leading to critical illness, an integer score from 0 to 4 was assigned to each categorical level such that a higher score was associated with a higher frequency. Then, we calculated the mean for each condition as the primary summary measure for the frequency at which that condition is reported. We used a similar approach for comparing the ease of accessibility to equipment, personnel, therapy, and other resources.

Utilizing the WFSICCM definition of three levels of ICUs, where Level 1 contains the most basic equipment and resources required to have a functional ICU and Level 3 has the most advanced [14], we matched each criterion with an appropriate survey response. We tallied the variables that hospitals reported as having access to “often” or “always” and counted those as having met those criteria. We then evaluated each criterion (across all levels) for each response in the data. Finally, we calculated several summary statistics based on these results, including the count and percentage of completed criteria for each level and hospital, as well as the cumulative count and percentage of completed criteria across all levels. We also calculated the number of hospitals that satisfied each respective criterion, in order to evaluate which critical care resources were more (or less) readily available.

We performed all statistical analysis using the R statistical programming language, version 3.4.4.

## Results

Of 53 health facilities identified and 48 with accurate contact information, 38 (79.2%) from all ten Haitian administrative departments completed the survey. Facility demographics are shown in Table 1. All facilities, including those that reported no ICU, reported caring for critically ill patients with a median of 12 (IQR 6–50) critically ill patients per week. Of hospitals without an ICU, 19 (82.6%) care for critically ill patients in the ED, five (21.7%) in a general medical ward, two (8.7%) in a postoperative ward, and four (17.4%) in other areas. For all facilities, only eight (21.1%) reported formal emergency or critical care training for physicians, and only five (13.2%) for nurses.

**Table 1:** Key demographic descriptors for all responding facilities (N = 38). ED = Emergency department. ICU = Intensive Care Unit. Neonates defined as <28 days old, children as 28 days to 13 years, adolescents as 14–18 years, adults as >18 years.

Category	N per region (%)	Category	N (%)
**Department**		**Total Beds**	
Centre	3 (7.9)	<50	11 (28.9)
Grand Anse	2 (5.3)	51–100	11 (28.9)
L’Artibonite	4 (10.5)	101–200	10 (26.3)
Nippes	2 (5.3)	201–300	5 (13.2)
Nord	3 (7.9)	301–400	0 (0.0)
Nord-est	1 (2.6)	>400	1 (2.6)
Nord-ouest	1 (2.6)		
Ouest	18 (47.4)	**Facility Language(s)**	
Sud	3 (7.9)	Haitian Creole	38 (100.0)
Sud-est	1 (2.6)	French	33 (86.8)
		English	12 (31.6)
**Payer Type**		Spanish	7 (18.4)
Private	10 (26.3)	Other	1 (2.6)
Not-for-profit	11 (28.9)		
Public Other	14 (36.8) 3 (7.9)	Critically Ill Patient Type(s)*	
		Neonates	28 (73.7)
**ED Present**		Children	29 (76.3)
Yes	36 (94.7)	Adolescents	30 (78.9)
No	2 (5.1)	Adults	35 (92.1)
**ICU Present**		**Medical Record**	
Yes	15 (38.5)	Paper	28 (73.7)
No	23 (60.5)	Electronic	12 (31.6)

* Patient types evaluated at any time across all responding facilities

There were 124 reported ICU beds nationally. Facilities without ICUs reported an additional 53 beds designated for critically ill patients, most of which were in emergency departments. The respondents reported the capability to mechanically ventilate a total of 62 patients within ICUs, and an additional six patients in other areas, not including operating theaters. Of the 15 facilities that reported having ICUs, the median number of patients that could be mechanically ventilated was similar to the number that could be noninvasively ventilated (Table 2), although the distribution of the two resources between hospitals was not symmetric.

**Table 2:** Key resource descriptors for hospitals reporting an ICU (N = 15). Values are N (%), unless otherwise stated. WFSICCM = World Federation of Societies of Intensive and Critical Care Medicine. Physician 24h = Physician available for ICU patients 24 hours per day. NIPPV = Non-invasive positive pressure ventilation.

Category	N (%)	Category	N (%)
**Payer Type**		**Physician Training**	
Private	4 (26.7)	Formal	5 (33.3)
Not-for-profit	5 (33.3)	Informal	3 (20.0)
Public	6 (40.0)	Basic	3 (20.0)
		Other	4 (26.7)
**WFSICCM Level**			
Level 1	5 (33.3)	**Nurse Training**	
Level 2	0 (0.0)	Formal	4 (26.7)
Level 3	0 (0.0)	Informal	3 (20.0)
Does not meet level	10 (66.7)	Basic	4 (26.7)
		Other	4 (26.7)
**Physician 24h**			
Yes	5 (33.3)		
No	10 (66.7)		
	**Min**	**Median (IQR)**	**Max**
**Critical Patients/Week**	2	12 (7.0–50.0)	200
**ICU Beds**	2	9 (3.5–10.5)	20
**Mechanical Ventilators**	0	3 (2.0–6.0)	11
**NIPPV**	0	3 (2.0–6.0)	10

Approximately one-third of the facilities reported around-the-clock physician availability for the ICU. Only one-third of facilities with ICUs reported formal critical care training for their physicians. Using the WFSICCM definition of an ICU (S1 Table), only five facilities met criteria for a Level 1 ICU, whereas none reached Levels 2 or 3. The remaining ten facilities with self-reported ICUs most commonly lacked increased nursing ratios, physicians trained in advanced cardiac or trauma life support, and noninvasive or invasive ventilation. For hospitals without an ICU, the most common missing criteria needed to achieve the WFSICCM definition for a Level 1 ICU can be found in Table 3.

**Table 3:** Presence of ICU-defining characteristics, according to the WFSICCM criteria, in Haitian hospitals caring for critically ill patients. IV = Intravenous; ATLS = Advanced trauma life support; RBCs = Red blood cells; BP = Blood pressure.

Criteria	Overall (N = 38)	Hospitals with ICU (N = 15)	Hospitals without ICU (N = 23)
**Satisfy All Level 1 Criteria (N, %)**	5, 13.2%	5, 33.33%	0, 0%
**Proportion of Satisfied Criteria (min, median, max)**	0.40, 0.70, 1.00	0.40, 0.90, 1.00	0.40, 0.70, 0.80
**Most Commonly Satisfied Criteria (N, %)**	-Antibiotics (IV) (37, 97.4%) -Bedside nurse daily (36, 94.7%) -Peripheral IV catheters (36, 94.7%)	-Antibiotics (IV) (14, 93.3%) -Bedside nurse daily (14,93.3%) -Nursing ratio ≥ 1:4 (14, 93.3%) -Automated vital signs (14, 93.3%) -Pulse oximetry (14, 93.3%) -Oxygen (any kind) (14, 93.3%)	-Antibiotics (IV) (23, 100%) -Peripheral IV catheters (23, 100%) -Bedside nurse daily (22, 95.7%)
**Least Commonly Satisfied Criteria (N, %)**	-Nursing ratio ≥ 1:4 (22, 57.9%) -Doctor ATLS training (15, 49.5%) -Non-invasive ventilation (12, 31.6%)	-EKG (12, 80.0%) -Non-invasive ventilation (10, 66.7%) -Doctor ATLS training (9, 60.0%)	-Nursing ratio ≥ 1:4 (8, 34.8%) -Doctor ATLS training (6, 26.1%) -Non-invasive ventilation (2,8.7%)
			
**Satisfy All Level 2 Criteria (N, %)**	0, 0%	0, 0%	0, 0%
**Proportion of Satisfied Criteria (min, median, max)**	0.00, 0.46, 0.92	0.23, 0.62, 0.92	0.0000, 0.31, 0.62
**Most Commonly Satisfied Criteria (N, %)**	-IV vasoactive drugs (28, 73.7%) -Pharmacist (25, 65.8%) -Nurse crit care training (22, 57.9%)	-IV vasoactive drugs (14, 93.3%) -Nursing ratio ≥ 1/3 (14, 93.3%) -Arterial blood gas (13, 86.7%)	-IV vasoactive drugs (14, 60.9%) -Pharmacist (14,60.9%) -Packed RBCs (13, 56.5%)
**Least Commonly Satisfied Criteria (N, %)**	-Arterial catheter (10, 26.3%) -Microbiologist (4, 10.5%) -Dialysis (any kind) (1, 2.6%)	-Arterial catheters (6, 40.0%) -Microbiologist (3, 20.0%) -Dialysis (any kind) (0, 0.0%)	-Mechanical ventilator (1, 4.3%) -Microbiologist (1, 4.3%) -Dialysis (any kind) (1, 4.3%)
			
**Satisfy All Level 3 Criteria (N, %)**	0, 0%	0, 0%	0, 0%
**Proportion of Satisfied Level 3 Criteria (min, median, max)**	0.08, 0.31, 0.77	0.15, 0.54, 0.69	0.08, 0.23, 0.54
**Most Commonly Satisfied Criteria (N, %)**	-Accepts transfers (25, 65.8%) -Automated BP cuff (25, 65.8%) -Ultrasound (22, 57.9%)	-Ultrasound (13, 86.7%) -Portable X-ray (12, 80.0%) -Automated BP cuff (12, 80.0%) -Nursing ratio ≥ 1/2 (12, 80.0%)	-Accepts transfers (15, 65.2%) -Automated BP cuff (13, 56.5%) -Platelets (11, 47.8%)
**Least Commonly Satisfied Criteria (N, %)**	-Doctor crit care training (7, 18.4%) -Respiratory therapist (7, 18.4%) -Participate in research (4, 10.5%) -Negative pressure isolation (3, 7.9%)	-Doctor crit care training (5, 33.3%) -Platelets (5, 33.3%) -Participate in research (3, 20.0%) -Negative pressure isolation (0, 0.0%)	-Doctor crit care training (2, 8.7%) -Participate in research (1, 4.3%) -Respiratory therapist (1, 4.3%)

**Fig 2:**
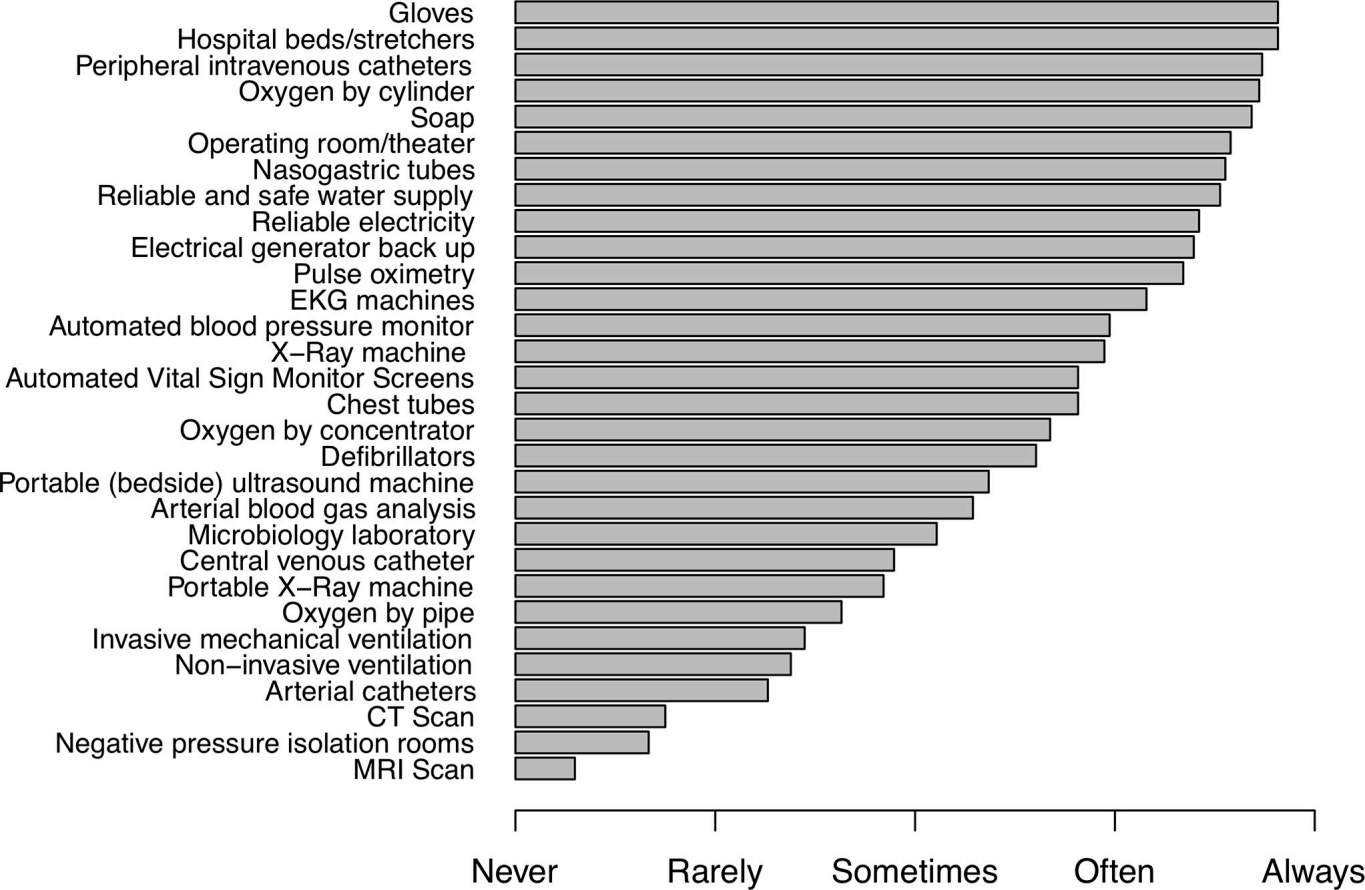
Availability of critical care equipment amongst all survey respondents (N = 38).

The most important reported barriers to providing critical care services included lack of portable x-ray machines, cardiac monitors, ventilators, trained emergency or critical care physicians, and physical space for ICUs. Availability of ICU-specific equipment is shown in Fig 2. Availability of personnel, ICU-specific therapies and other materials is shown in S1, S2 and S3 Figs. Specifically, 23 (60.5%) reported access to bedside ultrasound, while only seven (18.4%) reported access to CT scan and three (7.9%) to MRI. Additionally, 12 (31.6%) reported receiving donations they were unable to use. The majority of respondents (34, 89.5%) reported not conducting research. The distribution of disease states reported to cause critical illness in Haiti can be seen in S4 Fig.

## Discussion

The major finding of our nationwide survey of Haitian critical care is that current capacity remains insufficient to meet need. A significant, country-wide demand for critical care services exists at baseline, with regional demand likely increasing significantly during natural disasters and epidemics. The general paucity of ICU beds, equipment, professional training, and material resources is similar to findings from other LMICs. For a country of over 10 million people, our study estimates a total of 124 ICU beds. By comparison to other low-income countries, Uganda has 10 beds and Nepal has 167 beds per 10 million people [1], whereas Sri Lanka (a lower middle-income country) has 250 beds [15], South Africa (an upper middle-income country) has 957 beds [16] and the United States (a high-income country) has 3,125 beds per 10 million people [17, 18]. Unfortunately, there is no “magic number” of ICU beds to provide optimal critical care services, as diverse economic, cultural, and political values shape national and regional opinions on the benefits of—and even the very necessity of—critical care services. For low-income countries, such as Haiti, much more national-level data are required to help inform political and financial stakeholders, who must weigh innumerable competing priorities before allocating scarce funding to critical care services, which many consider expensive.

The major self-identified barriers to providing more effective critical care services in Haiti include lack of physical space to house critically ill patients, lack of basic critical care equipment, and few formally trained physicians and nurses. Only 37 of the 124 ICU beds meet the most basic (i.e., WFSICCM Level 1 ICU) international consensus standards of an ICU, highlighting the reality that even the most advanced ICUs in Haiti lack some of the most basic elements for intensive care delivery. Our findings bear great similarities to the only other known national-level acute care capacity study conducted in Haiti in 2014 [5]. In this trauma-specific cross-sectional audit of six tertiary and six secondary hospitals representing all 10 Haitian administrative departments, investigators highlighted significant limitations in equipment availability, personnel and training. Only three ICUs (defined as having access to mechanical ventilation and continuous monitoring) were identified and there were notable shortages of airway equipment, oxygen and pulse oximetry throughout the facilities. Only one-quarter of facilities had ATLS-trained physicians. Investigators similarly commented on the lack of a national continuing trauma education program. Despite the many limitations identified in our and prior studies, multiple investigations from LMIC settings indicate that streamlining critical care services through protocol development, education, and training can improve important clinical outcomes [19, 20].

Another major finding is that a significant amount of critical care in Haiti is provided outside of the ICU. This finding is similar to those from other LMIC settings [21] and reflective of the relative novelty of critical care as a distinct clinical entity in LMICs. The fact that over 80% of critically ill patients in Haitian hospitals without ICUs are cared for in the Emergency Department speaks to the important overlap between emergency and critical care medicine in LMICs. This overlap elucidates a fundamental conundrum facing the global critical care movement: should LMIC facilities devote significant constrained resources to developing ICU capacity specifically, or should critical care and emergency medicine function together under the auspices of global acute care? Whereas the WHO promotes acute care [2], international critical care professional societies promote a narrower vision [22]. Individual academic partnerships between facilities in high-income and low-middle-income countries tend to be specialty-specific [23–25], further fragmenting already disjointed systems of care. Addressing this question on the regional, national, and international levels through an organized, multidisciplinary process is essential to ensure efficient, cost-effective, and equitable critical care services in LMICs. Such efforts are already under way. A collaboration, for example, between the International Federation of Emergency Medicine and the WHO’s Emergency and Trauma Care Programme is developing a white paper on the continuum of care for critically ill and injured patients in low-resource settings [26]. This white paper will have an impressive emphasis on early critical care and transitions between components of the health system (personal communication, Teri Reynolds).

Given that improved critical care outcomes are possible outside of the ICU [19; 27, 28] and given the dearth of ICU physician presence in Haiti and other LMICs, ramping up critical care education and training for non-ICU clinicians and nurses is crucial. Structured critical care training can reduce adult [29] and pediatric ICU mortality in LMICs [30–32]. While Haiti has no dedicated postgraduate critical care training program, its only existing Emergency Medicine residency program provides critical care instruction [33]. Augmenting and expanding such training nationally with structured didactics, simulations, and bedside teaching may help to overcome some of the barriers to critical care delivery identified in our survey. Additionally, developing formal critical care nursing training programs, as has been done successfully in other LMIC environments [34], will mitigate inadequate numbers of ICU physicians. Haitian nurses have expressed a strong appetite for increased critical care-specific training [35] and capitalizing on this interest will go far to improve critical care capacity and outcomes in Haiti.

This study has some significant limitations. First, no single “official” hospital list contained a comprehensive, up-to-date list of active health facilities in Haiti. Due to the dynamic nature of Haitian health care, it is plausible that some facilities were missed. Moreover, facilities may have opened or closed since completion of our data collection, so our results should be interpreted as estimates rather than as concrete figures. Second, we did not achieve 100% survey penetration, so it is possible that some Haitian critical care resources remain unaccounted for. Third, surveys often were completed by medical directors who may play more of an administrative rather than clinical role and who may overstate or understate resource availability. A formal audit would be required to verify survey responses and to provide a more definitive accounting of critical care capacity. Despite these limitations, our study represents an important first step in establishing a national accounting of material and human resources for critical care in Haiti and identifying the practical needs of clinicians caring for critically ill patients across the country.

## Conclusions

The global critical care movement is rapidly advancing knowledge of the epidemiology and outcomes of critically ill patients in LMICs. To date, no comprehensive evaluation of critical care in Haiti has been performed. This study provides an important first look at critical care capacity in Haiti and establishes a baseline from which academicians, health facilities, and governmental and non-governmental organizations can coordinate an integrated and organized approach moving forward.

## Supporting information

S1 Table World Federation of Societies of Intensive and Critical Care Medicine’s (WFSICCM) proposed definition of an intensive care unit. IV = Intravenous. ATLS = Advanced trauma life support. BiPAP = Bilevel positive airway pressure. CPAP = continuous positive airway pressure. Adapted from Marshall JC, Bosco L, Adhikari NK et al. *J Crit Care*. 2017 Feb;37:270–276. Used with permission.
(DOCX)

S1 Fig Availability of ICU personnel amongst all facilities (N = 38). (DOCX)

S2 Fig Availability of ICU-specific therapies amongst all facilities (N = 38). (DOCX)

S3 Fig Availability of other ICU-relevant materials amongst all facilities (N = 38). (DOCX)

S4 Fig Distribution of disease states leading to critical illness amongst all facilities (N = 38). (DOCX)

S1 File Appendix 1. Facility List.
(DOCX)

S2 File Appendix 2. French version of survey instrument.
(DOCX)

S3 File Appendix 3. English version of survey instrument.
(DOCX)

S1 Dataset (XLSX)

## References

[1] Murthy S, Leligdowicz A, Adhikari NK. Intensive Care Unit Capacity in Low-Income Countries: A Systematic Review. *PLoS One*. 2015; 10(1): e0116949 10.1371/journal.pone.0116949 25617837 PMC4305307

[2] Hirshon JM, Risko N, Calvello EJ, Stewart de Ramirez S, Narayan M, Theodosis C, et al Health systems and services: the role of acute care. *Bull World Health Organ*. 2013 5 1;91(5):386–8. 10.2471/BLT.12.112664 23678202 PMC3646345

[3] World Bank. Haiti Overview. http://www.worldbank.org/en/country/haiti/overview. Accessed October 4, 2018.

[4] World Health Organization. Haiti: Country Profile. http://www.who.int/hac/crises/hti/background/profile/en/. Accessed October 4, 2018.

[5] McCullough C, DeGennaro V Jr, Bagley JK, Sharma J, Saint-Fort M, Henrys JH. A national trauma capacity assessment of Haiti. *J Surg Res*. 2016 3;201(1):126–33. 10.1016/j.jss.2015.10.018 26850193

[6] Dewberry L, McCullough C, Goss J, Hugar LA, Dente CJ, Sharma J. Trauma capacity in the central plateau department of Haiti. *J Surg Res*. 2014 11;192(1):34–40. 10.1016/j.jss.2014.06.009 25015749

[7] De Wulf A, Aluisio AR, Muhlfelder D, Bloem C. Emergency Care Capabilities in North East Haiti: A Cross-sectional Observational Study. *Prehosp Disaster Med*. 2015 12;30(6):553–9. 10.1017/S1049023X15005221 26487267

[8] Ministere de la Sante Publique et de la Population (MSPP). Liste Des Institutions Sanitaires Du Pays. April 2015. https://mspp.gouv.ht/site/downloads/Liste%20des%20Institutions%20Sanitaires%202015.pdf. Accessed January 5, 2017.

[9] Pan American Health Organization-World Health Organization health facilities database for Haiti. March, 2010. https://www.paho.org/disasters/index.php?option=com_content&view=article&id=1128:hospital-database&Itemid=0&lang=en. Accessed, January 5, 2017.

[10] United States Embassy in Haiti. Medical Assistance. February 2014. https://photos.state.gov/libraries/haiti/231771/PDFs/2014-3%20ACS%20hospitals%20list.pdf. Accessed January 5, 2017.

[11] Wikipedia. List of hospitals in Haiti. https://en.wikipedia.org/wiki/List_of_hospitals_in_Haiti. Accessed January 6, 2017.

[12] Haiti Christianity. Medical facilities. http://www.haitichristianity.org/about-haiti/medical-facilities. Accessed January 11, 2017.

[13] Westcott M, Martiniuk AL, Fowler RA, Adhikari NK, Dalipanda T. Critical care resources in the Solomon Islands: a cross-sectional survey. *BMC Int Health Hum Rights*. 2012 3 1;12:1 10.1186/1472-698X-12-1 22376229 PMC3307438

[14] Marshall JC, Bosco L, Adhikari NK, Connolly B, Diaz JV, Dorman T, et al What is an intensive care unit? A report of the World Federation of Societies of Intensive and Critical Care Medicine. *J Crit Care*. 2017 2;37:270–276. 10.1016/j.jcrc.2016.07.015 27612678

[15] Haniffa R, De Silva AP, Iddagoda S, Batawalage H, De Silva ST, Mahipala PG, et al A cross-sectional survey of critical care services in Sri Lanka: a lower middle-income country. *J Crit Care*. 2014 10;29(5):764–8. 10.1016/j.jcrc.2014.04.021 24929445

[16] Naidoo K, Singh J, Lalloo U. A critical analysis of ICU/HC beds in South Africa: 2008–2009. *S Afr Med J*. 2013 9 3;103(10):751–3. 10.7196/samj.6415 24079628

[17] Halpern N, Pastores S. Critical care medicine beds, use, occupancy and costs in the United States: a methodological review. *Crit Care Med*. 2015 11;43(11):2452–9. 10.1097/CCM.0000000000001227 26308432 PMC5520980

[18] United States Census Bureau. Population of the United States. https://www.census.gov/popclock/ Accessed August 8. 2018

[19] Papali A, Eoin West T, Verceles AC, Augustin ME, Nathalie Colas L, Jean-Francois CH, et al Treatment outcomes after implementation of an adapted WHO protocol for severe sepsis and septic shock in Haiti. *J Crit Care*. 2017 10;41:222–228. 10.1016/j.jcrc.2017.05.024 28591678

[20] Baker T, Schell CO, Lugazia E, Blixt J, Mulungu M, Castegren M, et al Vital Signs Directed Therapy: Improving Care in an Intensive Care Unit in a Low-Income Country. *PLoS One*. 2015 12 22;10(12):e0144801 10.1371/journal.pone.0144801 eCollection 2015. 26693728 PMC4687915

[21] Rudd KE, Kissoon N, Limmathurotsakul D, Bory S, Mutahunga B, Seymour CW, et al The global burden of sepsis: barriers and potential solutions. *Crit Care*. 2018 9 23;22(1):232 10.1186/s13054-018-2157-z 30243300 PMC6151187

[22] Schultz MJ, Dunser MW, Dondorp AM, Adhikari NK, Iyer S, Kwizera A, et al Current challenges in the management of sepsis in ICUs in resource-poor settings and suggestions for the future. *Intensive Care Med*. 2017 5;43(5):612–624. 10.1007/s00134-017-4750-z 28349179

[23] Boniface KS, Raymond A, Fleming K, Scott J, Kerry VB, Haile-Mariam T, et al The Global Health Service Partnership's point-of-care ultrasound initiatives in Malawi, Tanzania and Uganda. *Am J Emerg Med*. 2018 8 27 pii: S0735-6757(18)30716-2. 10.1016/j.ajem.2018.08.065 [Epub ahead of print] 30185389

[24] Maskalyk J and Landes M. The Toronto-Addis Ababa academic collaboration: emergency medicine. *Ethiop Med J*. 2014 7;Suppl 2:45–8. 25546909

[25] Sherman CB, Carter EJ, Braendli O, Getaneh A, Schluger NW. The East African Training Initiative. A Model Training Program in Pulmonary and Critical Care Medicine for Low-Income Countries. *Ann Am Thorac Soc*. 2016 4;13(4):451–5. 10.1513/AnnalsATS.201510-673OC 26991950

[26] International Federation of Emergency Medicine. Critical care in emergency medicine special interest group. https://www.ifem.cc/about-us/special-interest-groups/critical-care-in-emergency-medicine-special-interest-group/. Accessed November 1, 2018.

[27] Jacob ST, Moore CC, Banura P, Pinkerton R, Meya D, Opendi P, et al Severe sepsis in two Ugandan hospitals: a prospective observational study of management and outcomes in a predominantly HIV-1 infected population. Promoting Resource-Limited Interventions for Sepsis Management in Uganda (PRISM-U) Study Group. *PLoS One*. 2009 11 11;4(11):e7782 10.1371/journal.pone.0007782 19907656 PMC2771355

[28] Urayeneza O, Mujyarugamba P, Rukemba Z, Nyiringabo V, Ntihinyurwa P, Baelani JI, et al Increasing Evidence-Based Interventions in Patients with Acute Infections in a Resource-Limited Setting: A Before-and-After Feasibility Trial in Gitwe, Rwanda; Sepsis in Resource-Limited Nations Workgroup of the Surviving Sepsis Campaign. *Crit Care Med*. 2018 8;46(8):1357–1366. 10.1097/CCM.0000000000003227 29957715

[29] Haniffa R, Lubell Y, Cooper BS, Mohanty S, Alam S, Karki A, et al Impact of a structured ICU training programme in resource-limited settings in Asia. *PLoS One*. 2017 3 14;12(3):e0173483 10.1371/journal.pone.0173483 eCollection 2017. 28291809 PMC5349661

[30] Campos-Mino S, Sasbón JS, Von Dessauer B. Pediatric intensive care in Latin America. *Med Intensiva*. 2012 Jan-Feb;36(1):3–10. 10.1016/j.medin.2011.07.004 21906846

[31] Nakachi G, Shimbaku R, Cieza J. Assessment of survival in a pediatric intensive care unit in Lima, Peru. *Internet J Emerg Intensive Med*. 2009 12(1). http://ispub.com/IJEICM/12/1/11541.

[32] Canarie MF, Shenoi AN. Teaching the Principles of Pediatric Critical Care to Non-Intensivists in Resource Limited Settings: Challenges and Opportunities. *Front Pediatr*. 2018 3 2;6:44 10.3389/fped.2018.00044 eCollection 2018. 29552547 PMC5840157

[33] Marsh RH, Rouhani SA, Pierre P, Farmer PE. Strengthening emergency care: experience in central Haiti. *Lancet Glob Health*. 2015 4 27;3 Suppl 2:S5–7. 10.1016/S2214-109X(14)70378-X 25926321

[34] De Silva AP, Stephens T, Welch J, Sigera C, De Alwis S, Athapattu P, et al Nursing intensive care skills training: a nurse led, short, structured, and practical training program, developed and tested in a resource-limited setting. *J Crit Care*. 2015 4;30(2):438.e7–11. 10.1016/j.jcrc.2014.10.024 25466312

[35] Losonczy LI, Williams S, Papali A, Costantino CA, Colas LN, Patel BM, et al Haiti Acute and Emergency Care Conference: descriptive analysis of an acute care continuing medical education program. *J Glob Health Rep* 2019; 3: e2019012 10.29392/joghr.3.e2019012

