# Supplementary material for: Critical care capacity in Haiti: A nationwide cross-sectional survey
Source: PLoS One. 2019 Jun 13;14(6):e0218141. doi: 10.1371/journal.pone.0218141 (PMC6565360; doi:10.1371/journal.pone.0218141)
Supplement: S1 Table — IV = Intravenous. ATLS = Advanced trauma life support. BiPAP = Bilevel positive airway pressure. CPAP = continuous positive airway pressure. Adapted from Marshall JC, Bosco L, Adhikari NK et al. J Crit Care. 2017 Feb;37:270–276. Used with permission. (DOCX) [file pone.0218141.s001.docx]

**S1 Table.** World Federation of Societies of Intensive and Critical Care Medicine’s (WFSICCM) proposed definition of an intensive care unit. IV = Intravenous. ATLS = Advanced trauma life support. BiPAP = Bilevel positive airway pressure. CPAP = continuous positive airway pressure. Adapted from Marshall JC, Bosco L, Adhikari NK et al. *J Crit Care.* 2017 Feb;37:270-276. Used with permission.

| **Level One** | **Level Two** | **Level Three** |
| --- | --- | --- |
| -Peripheral IV catheters   -Oxygen (any kind) -Antibiotics (IV)  -EKG  -Doctor ATLS Training  -Bedside nurse daily -Pulse oximetry -Vital signs monitor  -Non-invasive ventilation (BiPAP/CPAP) -ICU nursing ratio ≥ 1/4 | -Packed Red Blood Cells -Mechanical ventilation  -Dialysis (any kind) -Arterial blood gas -IV inotropes/vasopressors  -Doctor formal/ informal critical care training -Nurse formal/informal critical care training -Pharmacist -Microbiologist -Subspecialty Surgeon -ICU nursing ratio ≥ 1/3  -Central venous catheters -Arterial catheters | -Platelets -Fresh frozen plasma -Automated blood pressure  -Doctor formal/informal critical care training  -Nurse formal/informal critical care training  -Doctor present 24 hours/day  -Respiratory therapist  -Negative pressure isolation -Participate in research  -Accepts transfers from other hospitals  -ICU nursing ratio ≥1/2  -Portable X-ray -Ultrasound |
