# Supplementary figures and images for: Critical care capacity in Haiti: A nationwide cross-sectional survey
Source: PLoS One. 2019 Jun 13;14(6):e0218141. doi: 10.1371/journal.pone.0218141 (PMC6565360; doi:10.1371/journal.pone.0218141)

**S1 Fig.** Availability of ICU personnel amongst all facilities (N=38).


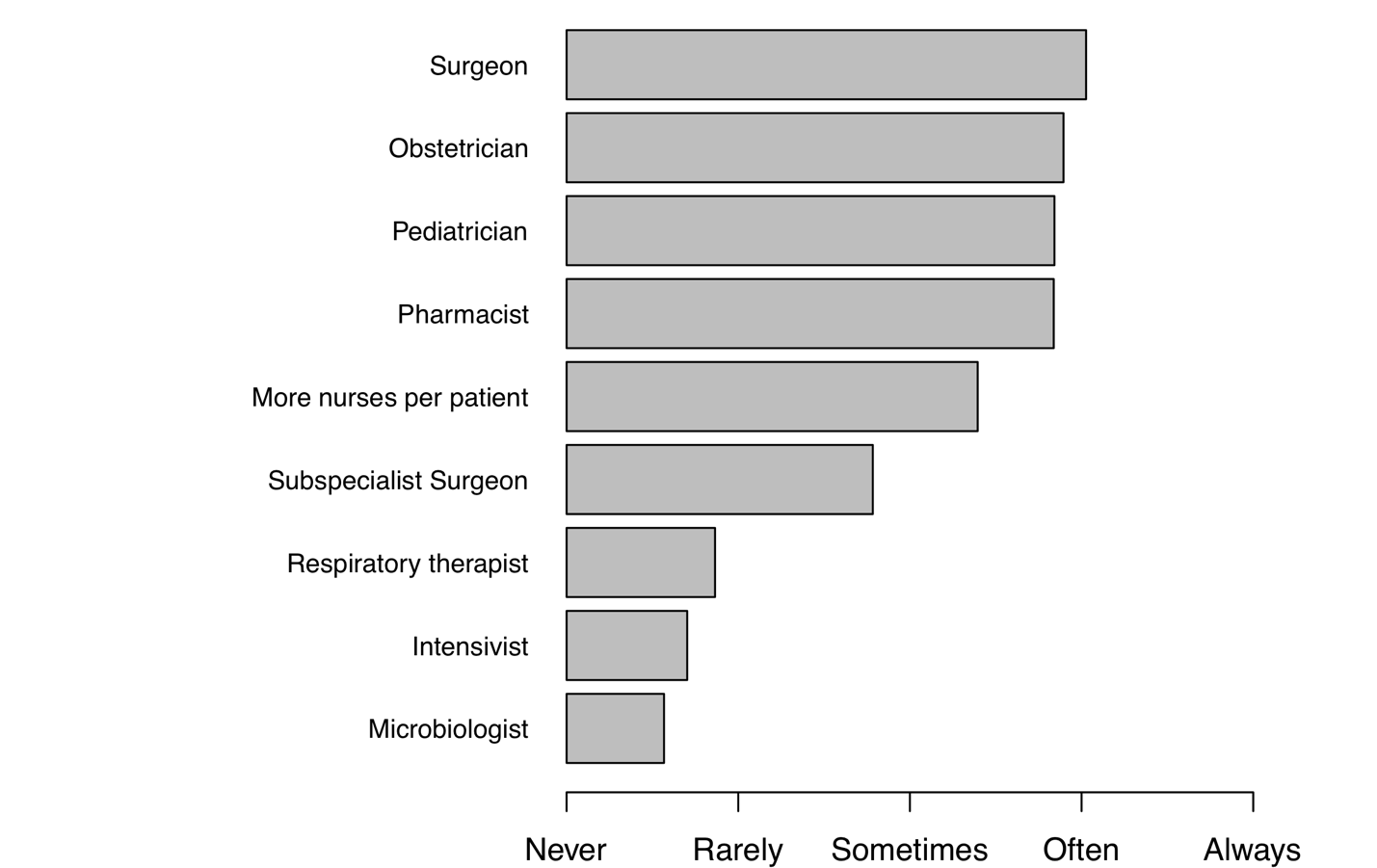

Supplement: S1 Fig — (DOCX) [file pone.0218141.s002.docx]

**S2 Fig.** Availability of ICU-specific therapies amongst all facilities (N=38).


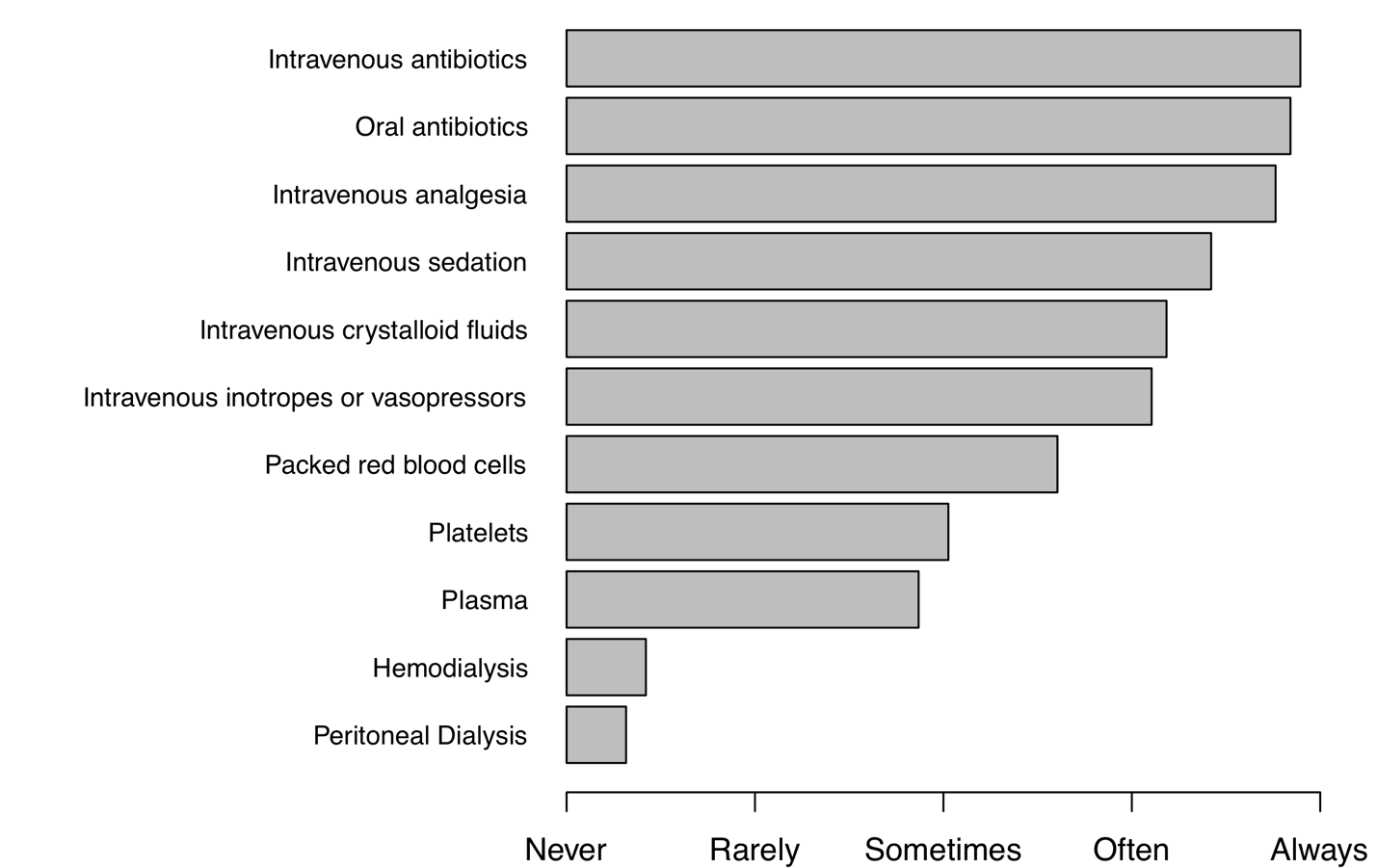

Supplement: S2 Fig — (DOCX) [file pone.0218141.s003.docx]

**S3 Fig.** Availability of other ICU-relevant materials amongst all facilities (N=38).


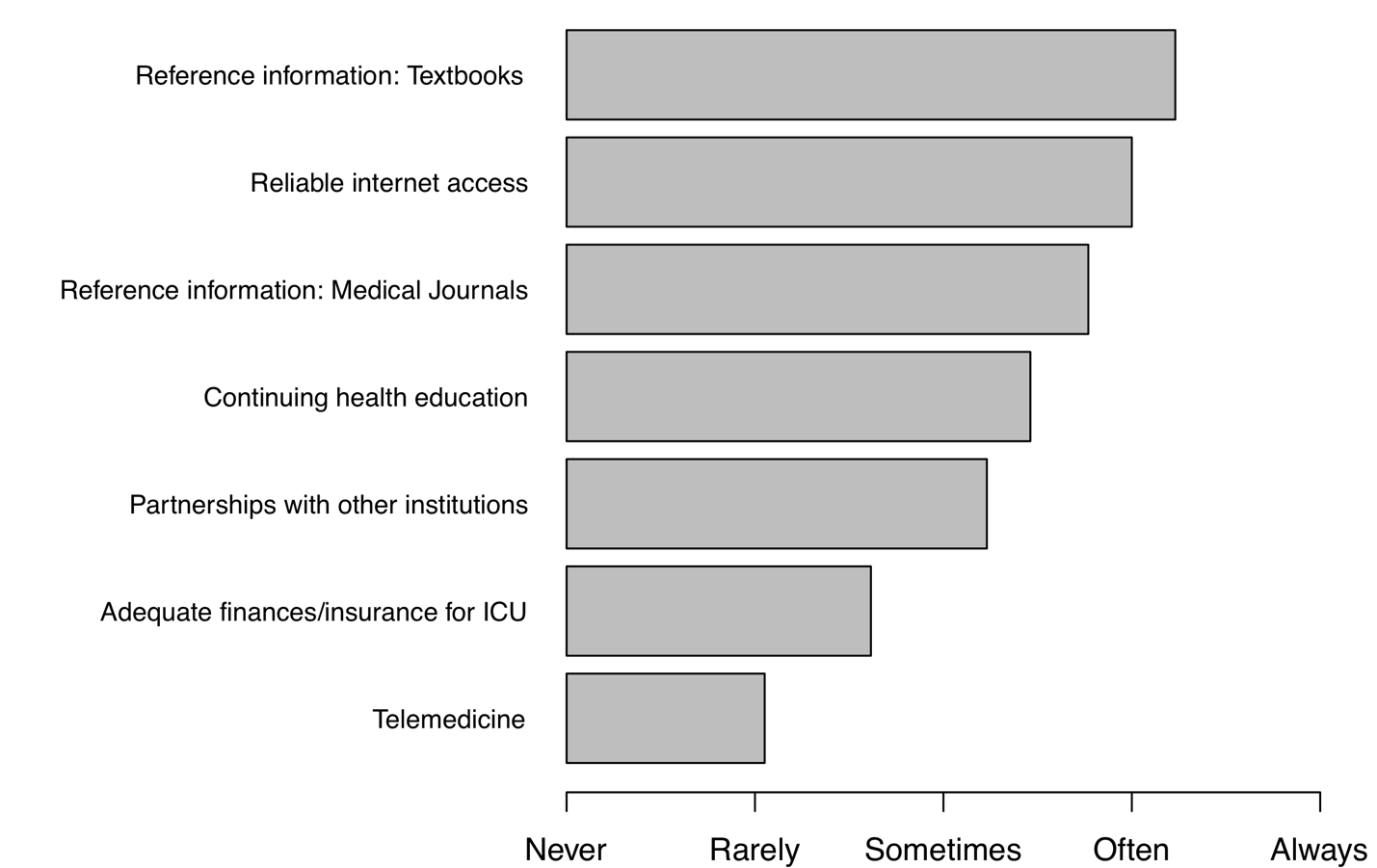

Supplement: S3 Fig — (DOCX) [file pone.0218141.s004.docx]

**S4 Fig.** Distribution of disease states leading to critical illness amongst all facilities (N=38)


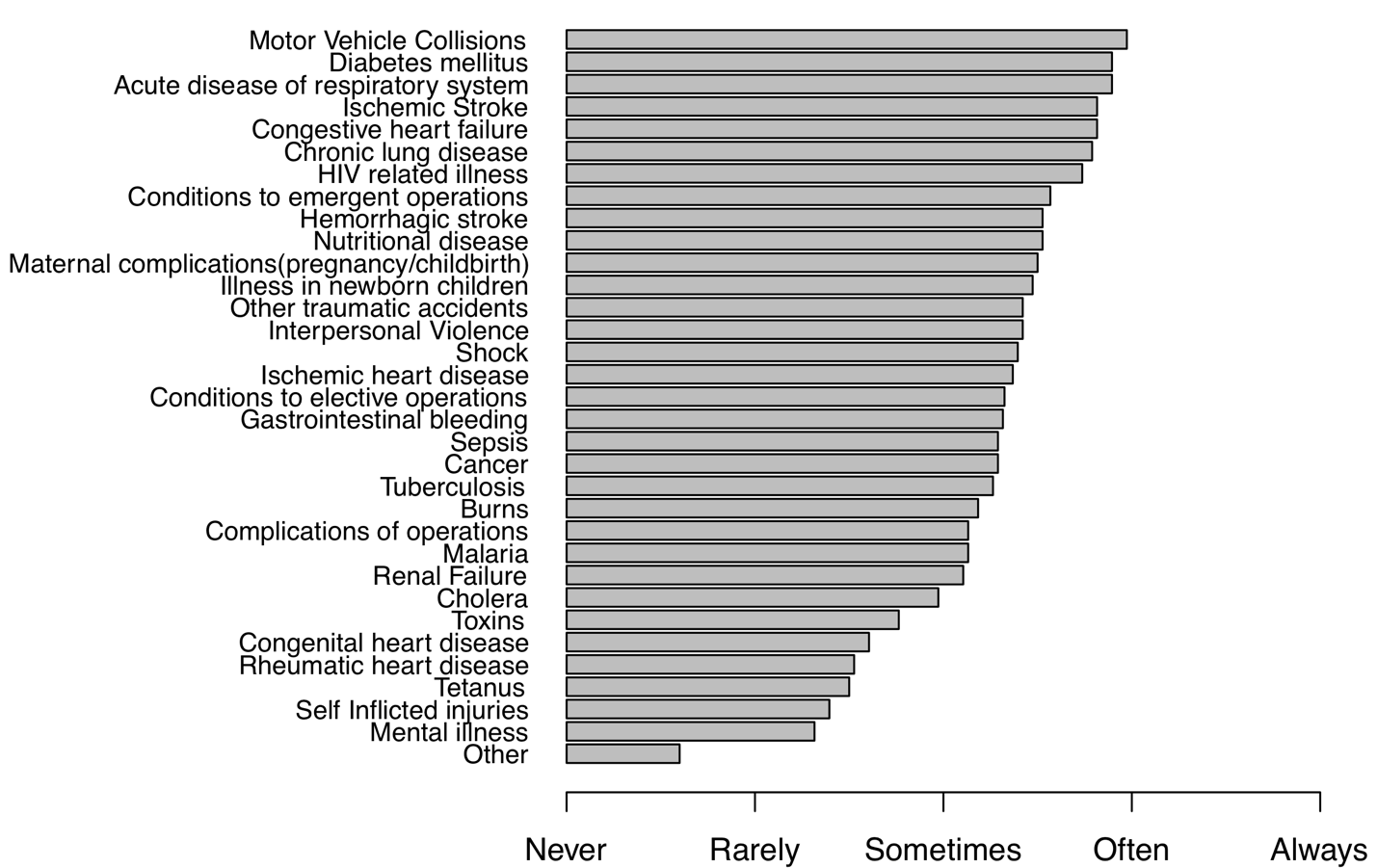

Supplement: S4 Fig — (DOCX) [file pone.0218141.s005.docx]
