# Supplementary material for: Critical care capacity in Haiti: A nationwide cross-sectional survey
Source: PLoS One. 2019 Jun 13;14(6):e0218141. doi: 10.1371/journal.pone.0218141 (PMC6565360; doi:10.1371/journal.pone.0218141)
Supplement: S1 File — Facility List. (DOCX) [file pone.0218141.s006.docx]

**S1 File—Appendix 1.** Facility List.

| **Department** | **Facility** | **Contacted** | **Survey Response** |
| --- | --- | --- | --- |
| Ouest |  |  |  |
|  | -Hôpital Bernard Mevs  -Hôpital de l’Université d’Etat d’Haiti (HUEH)  -Hôpital OFATMA Cite-Militaire  -Hôpital St. Damien (pediatric only)  -Hôpital Universitaire La Paix  -Hôpital St. Luc  -Hôpital Espoir  -Hôpital Canapé-vert  -Hôpital Petit-Goave/Hôpital Notre Dame  -Hôpital Adventiste, Port-au-Prince  -Hôpital Saint-François de Sales  -Hôpital Grâce pour Enfants, Port-au-Prince  -Hôpital Eliazar Germaine  -Chirurgicale Lambert Sante  -MSF Tabarre (Chirurgie et Traumato)  -Hôpital de Fermathe  -Foyer Ste Camille,  -Hôpital City Med de Petionville | Yes | Yes |
|  | -COPASSA  -Hôpital St. Louis  -Hôpital Le Messie  -Hôpital Communautaire de Bon Repos  -MSF Brules | Yes | No |
|  | -Hôpital Haiti Health Ministries | N/A (Clinic only) | N/A |
|  | -Hôpital Français D’Haiti 378  -Hôpital Asile Français | No (No contact info) | N/A |
|  |  |  |  |
| Nord | -Hôpital Universitaire Justinien, Cap-Haïtien  -Hôpital Sacré-Coeur CRUDEM Milot  -OFATMA Cap Haitien | Yes | Yes |
|  | -Convencion Baptist D’Haiti  -CAL Grande Riviere du Nord, Grande-Riviere-HCR | No (No contact info) | N/A |
|  |  |  |  |
|  |  |  |  |
| L'Artibonite | -Hôpital Albert Schweitzer, Deschapelles  -Hôpital La Providence, Gonaïves  -Hôpital Bienfaisance de Pignon  -Hôpital Saint Nicolas, Saint Marc | Yes | Yes |
|  | -Hôpital Charles Colimon (Petite Riviere)-PIH  -Hôpital Dumarsais Estime (verettes)-PIH | Yes | No |
|  |  |  |  |
| Nord-Ouest | -Hôpital de Immaculée Conception, Port-de-Paix | Yes | Yes |
|  | -Hôpital Convention Baptiste | Yes | No |
|  |  |  |  |
| Nord-est | -Hôpital de Fort-Liberte, Fort-liberte, HD | Yes | Yes |
|  | -Univers Medical Center, Ouanaminthe | N/A (clinic only) | N/A |
|  |  |  |  |
| Sud-est | -Centre Hospitalier Christian Martinez | Yes | Yes |
|  | -Hôpital Saint-Michel de Jacmel | Yes | No |
|  | -Hôpital Platon Besace | No (No contact info) | N/A |
|  | -Clinic Bas Grandoux | N/A (clinic only) | N/A |
|  |  |  |  |
| Nippes | -Hôpital Sainte-Thérèse de Miragoâne  -Hôpital de Référence Communautaire de l’Asile | Yes | Yes |
|  |  |  |  |
| Sud | -Hôpital Immaculée Conception, Les Cayes  -Hôpital St. Boniface  -Hôpital Notre Dame des Cayes | Yes | Yes |
|  | -Hôpital OFATMA Les Cayes | Yes | No |
|  |  |  |  |
| Centre | -Hôpital Sainte-Thérèse de Hinche-PIH/ZL  -Hôpital Universitaire de Mirebalais  -Hôpital Bon Sauveur | Yes | Yes |
|  |  |  |  |
| Grand'Anse | -Hôpital Sainte-Antoine de Jérémie  -Hôpital Dame-Marie, Dame-Marie | Yes | Yes |
|  |  |  |  |
|  |  |  |  |
| **Totals** | Departments: 10 of 10 |  |  |
|  | Number facilities meeting criteria: 53 |  |  |
|  | Number facilities contacted: 48 |  |  |
|  | Number facilities uncontacted: 5 |  |  |
|  | Survey responses: 38 |  |  |
|  |  |  |  |
|  |  |  |  |
