# Supplementary material for: Critical care capacity in Haiti: A nationwide cross-sectional survey
Source: PLoS One. 2019 Jun 13;14(6):e0218141. doi: 10.1371/journal.pone.0218141 (PMC6565360; doi:10.1371/journal.pone.0218141)
Supplement: S2 File — French version of survey instrument. (DOCX) [file pone.0218141.s007.docx]

**S2 File—Appendix 2.** French version of survey instrument.

**Enquête globale sur les ressources disponibles aux soins des patients gravement malades**

Cher/chère collègue,

Nous vous envoyons ce sondage dans le bût de mieux comprendre les maladies graves en Haïti. Ce sondage recueille des données au sujet de vous, votre environnement de travail, ainsi que les ressources dont vous avez à votre disposition pour traiter les patients gravement malades. Vos réponses éclairciront les soins intensifs en Haïti et votre participation est inestimable à cet égard.

Ce sondage prendra à peu près 15 à 20 minutes à compléter. En participant, votre consentement à l’utilisation de vos données est implicite. Vos réponses sont confidentielles. Veuillez répondre à votre connaissance, et n’hésitez pas de consulter vos collègues si vous êtes incertain(e) de vos réponses. Si vous êtes incapable de répondre à une des questions, veuillez ne rien y marquer.

Cette étude est coordonnée par la Fondation St. Luke à Port-au-Prince, Haïti et l’Université de Maryland à Baltimore, É.-U. et a été vérifiée par les comités d’éthiques de la recherche des deux institutions.

Nous apprécions beaucoup votre participation

1. **Information personnel**
2. Quel est votre rôle ? Veuillez sélectionner l’une des réponses suivantes :
   - Médecin
   - Infirmier(ière)
   - Administrateur(trice)
   - Autre (veuillez préciser) : __________________________
3. Quel est le niveau d’éducation le plus élevé que vous avez atteint ? Veuillez sélectionner l’une des réponses suivantes :
   - École secondaire
   - Formation professionnelle pour devenir agent clinique
   - Formation professionnelle pour devenir infirmier(ière)
   - Baccalauréat (diplôme en études de premier cycle universitaire en soins infirmiers ou médecine)
   - Formation en internat médical/chirurgical
   - Formation de souspécialité ou stage clinique postdoctoral
   - Autre (veuillez préciser): ___________________________________
4. Si vous êtes médecin, quelle est votre souspécialité ?

- Médecine interne
- Médecine d’urgence
- Chirurgie générale
- Anesthésie
- Obstétrique/gynécologie
- Soins intensifs
- Autre (veuillez préciser) : ____________________________________

1. Si vous êtes médecin, depuis combien de temps pratiquez-vous la médecine? Veuillez sélectionner l’une des réponses suivantes :
   - 0 à 5 ans
   - 6 à 10 ans
   - 11 à 15 ans
   - 16 à 20 ans
   - Plus que 20 ans
2. Travaillez-vous à plus qu’une institution ? Veuillez sélectionner l’une des réponses suivantes :
   - Oui
   - Non

Si vous avez répondu ‘oui’ à la question précédente, veuillez faire référence à l’institution où vous travaillez plus fréquemment en répondant aux questions suivantes.

1. **Information institutionnel**

Nom de l’institution : ________________________________________________________

Province/région : ___________________________________________________________

Ville/village : _______________________________________________________________

Code postal de l’institution s’il y a lieu : __________________________________________

Adresse de l’institution s’il y a lieu : ______________________________________________

Coordonnées téléphoniques de l’institution s’il y a lieu (incluant le code régional): ________

1. Sélectionnez le type d’institution où vous travaillez :
   - Hôpital académique affilié à une faculté de médecine
   - Hôpital de recours national non-affilié à une faculté de médecine
   - Hôpital régional
   - Hôpital général de district
   - Autre (veuillez préciser): ______________________
2. D’où proviennent les ressources financières de l’institution? Veuillez sélectionner toute(s) réponse(s) pertinente(s) :
   - Le gouvernement
   - Une organisation à but non lucratif (p. ex.: un organisme caritatif, une mission)
   - Une organisation à but lucratif (p. ex. : une corporation)
   - Les forces armées
   - Autre (veuillez préciser): ______________________
3. Est-ce que l’hôpital a une salle d’urgence ? Veuillez sélectionner l’une des réponses suivantes :
   - Oui
   - Non
4. Combien de lits y a-t-il dans votre institution ? _________________________________

Si vous ne connaissez pas la réponse exacte, veillez sélectionner l’une des réponses suivantes :

- - <50
  - 51-100
  - 101-200
  - 201-300
  - 301-400
  - >400

1. Est-ce que votre institution accepte des transferts de d’autres institutions ? Veuillez sélectionner l’une des réponses suivantes :
   - Jamais
   - Rarement
   - Parfois
   - Fréquemment
   - Très fréquemment
2. Est-ce que votre institution transfert des patients à une institution de recours ? Veuillez sélectionner l’une des réponses suivantes :
   - Jamais
   - Rarement
   - Parfois
   - Fréquemment
   - Très fréquemment
3. Quelle(s) langue(s) sont utilisées dans la provision des soins à votre institution ? Veuillez sélectionner toute(s) réponse(s) pertinente(s) :
   - Français
   - Créole
   - Anglais
   - Espagnol
   - Autre (veuillez préciser): _____________________
4. Comment est-ce que les soins sont documentés à votre institution ? Veuillez sélectionner toute(s) réponse(s) pertinente(s) :

- Dossier papier
- Dossier électronique/digital
- Combinaison de dossier papier et électronique
- Radiologie digitale
- Radiologie traditionnelle (avec films)
- Autre (veuillez préciser): _____________________

**Veuillez répondre aux questions suivantes au sujet de la partie de l’hôpital impliquée dans les soins des patients gravement malades (p. ex. : patients en choc, en détresse respiratoire, ou étant polytraumatisés).**

1. **Prise en charge des patients gravement malades**
2. Où sont les patients gravement malades soignés dans l’hôpital ? Veuillez sélectionner toute(s) réponse(s) pertinente(s) :
   - Unité de soins intensifs (USI étant un lieu dans l’hôpital désigné spécifiquement pour les patients gravement malades avec soins infirmiers supplémentaires)
   - Unité de récupération post-opératoire
   - Salle d’urgence
   - Unité de soins généraux
   - Unité de soins pour malades hautement dépendants /unité de soins aigus (unité intermédiaire entre une unité de soins généraux et une USI)
   - Autre (veuillez préciser): _____________________
3. Si votre hôpital a une unité de soins intensifs (USI), veuillez répondre aux questions suivantes :
4. Combien de lits sont disponibles dans cette unité ?

Veuillez indiquer le nombre de lits :_____________

1. Combien d’infirmiers travaillent pendant une période de travail typique ? Veuillez indiquer le nombre d’infirmiers :______________________
2. Y a-t-il un médecin présent physiquement dans l’unité 24h par jour ? Veuillez sélectionner l’une des réponses suivantes :
   - - Oui
     - Non
3. Si non, combien d’heures par jour est-ce que le médecin est présent dans l’USI ?
   - - 1 à 2 heures
     - 3 à 5 heures
     - 6 à 9 heures
     - 10 à 12 heures
     - 13 à 23 heures
     - 24 heures
4. En moyenne, quel pourcentage des lits est occupé chaque journée ? Veuillez sélectionner l’une des réponses suivantes :
   - - 0% à 25%
     - 26% à 50%
     - 51% à 75%
     - 76% à 100%
5. Combien de patients peuvent recevoir la ventilation mécanique par canule endotrachéale à la fois ?

Veuillez inscrire le nombre de patients : ____________________________

1. Combien de patients peuvent recevoir la ventilation mécanique non-invasive par masque à la fois  (soit la ventilation spontanée en pression positive (VSPPC) ou la ventilation spontanée en pression positive bidirectionnelle (VSPPBi))?

Veuillez inscrire le nombre de patients : _____________________________

1. Combien de patients gravement malades ou blessés est-ce que votre institution a soigné dans la dernière semaine ? Si vous avez une USI ou une unité de soins pour malades hautement dépendants, veuillez inclure les patients qui y ont été admis, ainsi que tous les patients gravement malades ou blessés qui ont été soigné hors de cette unité.

Veuillez inscrire le nombre de patients : ________________

1. Quelle tranche d’âge de patients gravement malades est soignée par votre institution ? Veuillez sélectionner toute(s) réponse(s) pertinente(s) :
   - Nouveau-nés (âgés <28 jours)
   - Enfants (âgés de 28 jours à 14 ans)
   - Adolescents (âgés de 15 à 18 ans)
   - Adultes (âgés > 18 ans)
2. Si vous soignez les adultes et les adolescents gravement malades, quel est l’âge moyen approximatif de TOUS les patients gravement malades que vous avez soigné dans le DERNIER MOIS ?
   - 15 à 18 ans
   - 19 à 29 ans
   - 30 à 49 ans
   - 50 à 69 ans
   - >70 ans
3. Si vous soignez les enfants gravement malades, quel est l’âge moyen approximatif de TOUS les enfants gravement malades que vous avez soigné dans le DERNIER MOIS ?
   - Nouveau-nés (âgés <28 jours)
   - 29 jours à 11 mois
   - 1 à 4 ans
   - 5 à 9 ans
   - 10 à 14 ans
4. Qui soigne les patients gravement malades au chevet QUOTIDIENNEMENT? Veuillez indiquer tous les professionnels de santé impliqués, même s’ils sont présents pour seulement une partie de la journée :

- Médecin (si présent, veuillez indiquer leur(s) souspécialité(s))
  - Anesthésie
  - Médecine interne
  - Pneumologie
  - Chirurgie
  - Soins intensifs
  - Pédiatrie
  - Généraliste/Omnipractien(ne)
  - Autre (veuillez préciser) : ______________________________
- Adjoint(e) au médecin (p. ex. : agent(e) clinique)
- Infirmier(ière)
- Thérapeute respiratoire
- Pharmacien(ienne)
- Famille ou amis du patient
- Autre personnel (veuillez préciser) :____________________________

1. Est-ce que les médecins qui soignent les patients gravement malades ont complété la/les formation(s) suivante(s) :
   - Formation officielle en soins intensifs (p. ex. : formation médicale postdoctorale)
   - Formation informelle en soins intensifs (p. ex. : expérience dans une unité de soins intensifs à l’étranger)
   - Cours en soins intensifs de base (p. ex. : soins avancés de réanimations des polytraumatisés (ATLS), soins avancés en réanimation cardiovasculaire (SARC), soins avancés en réanimation pédiatrique (SARP), BASIC, EDICS, étc.)
   - Autre (veuillez préciser) :_________________________
2. Est-ce que les infirmiers qui soignent les patients gravement malades ont complété la/les formation(s) suivante(s) :
   - Formation officielle en soins intensifs
   - Formation informelle en soins intensifs (p. ex. : expérience dans une unité de soins intensifs à l’étranger)
   - Cours en soins intensifs de base (p. ex. : soins avancés de réanimations des polytraumatisés (ATLS), soins avancés en réanimation cardiovasculaire (SARC), soins avancés en réanimation pédiatrique (SARP), BASIC, EDICS, étc.)
   - Autre (veuillez préciser) :_________________________

Avec quelle fréquence traitez-vous les patients atteints de maladie grave causée par chaque état dénommé ci-dessous? Veuillez choisir l’une des réponses suivantes par condition : jamais, rarement, parfois, souvent, ou toujours.

|  | **Jamais** | **Rarement** | **Parfois** | **Souvent** | **Toujours** |
| --- | --- | --- | --- | --- | --- |
| Accidents de la circulation routière |  |  |  |  |  |
| Complications maternelles de la grossesse et de l'accouchement |  |  |  |  |  |
| Brûlures |  |  |  |  |  |
| Toxines (p. ex.: empoisonnement, morsure de serpent, etc.) |  |  |  |  |  |
| Violence interpersonnelle (p. ex. : agression, homicide) |  |  |  |  |  |
| Blessures auto-infligées (p. ex. : tentative de suicide) |  |  |  |  |  |
| Autre traumatismes (p. ex. : chute d’un toit, noyade) |  |  |  |  |  |
| Maladies chez les nouveau-nés |  |  |  |  |  |
| Tuberculose |  |  |  |  |  |
| Paludisme |  |  |  |  |  |
| Tétanos |  |  |  |  |  |
| Maladies reliées au VIH |  |  |  |  |  |
| Insuffisance cardiaque congestive |  |  |  |  |  |
| Cardiopathie ischémique |  |  |  |  |  |
| Maladie cardiaque congénitale |  |  |  |  |  |
| Maladie cardiaque rhumatismale |  |  |  |  |  |
| Maladies nutritionnelles |  |  |  |  |  |
| Cancer |  |  |  |  |  |
| Maladies pulmonaires chroniques (p. ex. : asthme, MPOC) |  |  |  |  |  |
| Maladies aigües du système respiratoire (p. ex. : pneumonie, SDRA) |  |  |  |  |  |
| Accident cérébrovasculaire ischémique |  |  |  |  |  |
| Accident cérébrovasculaire hémorragique |  |  |  |  |  |
| Conditions médicales menant à une opération élective |  |  |  |  |  |
| Conditions médicales menant à une opération d’urgence |  |  |  |  |  |
| Complications post-opératoires |  |  |  |  |  |
| Septicémie |  |  |  |  |  |
| Insuffisance rénale |  |  |  |  |  |
| Diabète sucré |  |  |  |  |  |
| Saignement du tractus gastro-intestinal |  |  |  |  |  |
| Choléra |  |  |  |  |  |
| Choc |  |  |  |  |  |
| Maladie mentale |  |  |  |  |  |
| Autre 1 (Veuillez préciser) |  |  |  |  |  |
| Autre 2 (Veuillez préciser) |  |  |  |  |  |

k) Des conditions dénommées ci-dessus, veuillez classer et énumérer les 5 causes de décès principales des patients gravement malades que vous traitez à votre institution**:**

1) _____________________________________________

2) _____________________________________________

3) _____________________________________________

4) _____________________________________________

5) _____________________________________________

l) Quelles ressources sont disponibles pour soigner les patients gravement malades à votre institution? Veuillez choisir l’une des réponses suivantes par article : jamais, rarement, parfois, souvent, ou toujours.

| Personnel | **Jamais** | **Rarement** | **Parfois** | **Souvent** | **Toujours** |
| --- | --- | --- | --- | --- | --- |
| Plus d’infirmiers(ières) par patient que dans une unité de soins généraux |  |  |  |  |  |
| Pédiatre |  |  |  |  |  |
| Obstétricien(ne) |  |  |  |  |  |
| Chirurgien(ne) |  |  |  |  |  |
| Thérapeute respiratoire |  |  |  |  |  |
| Pharmacien(ienne) |  |  |  |  |  |
| Chirurgien(ne) ayant une formation en surspécialité (p. ex. : neurochirurgien(ne), otorhinolaryngologiste, urologue) |  |  |  |  |  |
| Intensiviste |  |  |  |  |  |
| Microbiologiste |  |  |  |  |  |

| Équipement | **Jamais** | **Rarement** | **Parfois** | **Souvent** | **Toujours** |
| --- | --- | --- | --- | --- | --- |
| Lits d'hôpitaux /civières |  |  |  |  |  |
| Bloc opératoire |  |  |  |  |  |
| Approvisionnement fiable d’électricité |  |  |  |  |  |
| Alternateur de secours |  |  |  |  |  |
| Approvisionnement fiable et sûre d’eau |  |  |  |  |  |
| Savon |  |  |  |  |  |
| Gants |  |  |  |  |  |
| Source d’oxygène : Oxygénoduc |  |  |  |  |  |
| Source d’oxygène : Bonbonne d’oxygène |  |  |  |  |  |
| Source d’oxygène: Concentrateur d’oxygène |  |  |  |  |  |
| Moniteur de saturation d’oxygène |  |  |  |  |  |
| Ventilation mécanique non-invasive (VSPPC, VSPPBi) |  |  |  |  |  |
| Ventilation mécanique invasive |  |  |  |  |  |
| Tubes nasogastriques |  |  |  |  |  |
| Drains thoraciques |  |  |  |  |  |
| Analyse de sang artériel |  |  |  |  |  |
| Électrocardiographe |  |  |  |  |  |
| Tensiomètre automatisé |  |  |  |  |  |
| Moniteurs de signes vitaux automatisés |  |  |  |  |  |
| Défibrillateur cardiaque |  |  |  |  |  |
| Appareil de radiographie (non-portable) |  |  |  |  |  |
| Appareil de radiographie portable |  |  |  |  |  |
| Appareil à ultrason portable |  |  |  |  |  |
| Tomodensitomètre (TDM) |  |  |  |  |  |
| Imagerie par résonance magnétique (IRM) |  |  |  |  |  |
| Chambres d’isolation à pression négative |  |  |  |  |  |
| Laboratoire microbiologique |  |  |  |  |  |
| Cathéters artériels |  |  |  |  |  |
| Cathéters intraveineux centraux |  |  |  |  |  |
| Cathéters intraveineux périphériques |  |  |  |  |  |

| Thérapie | **Jamais** | **Rarement** | **Parfois** | **Souvent** | **Toujours** |
| --- | --- | --- | --- | --- | --- |
| Fluides cristalloïdes par voie intraveineuse |  |  |  |  |  |
| Médicaments de soutien cardio-vasculaire par voie intraveineuse (vasopresseurs, ionotropes) |  |  |  |  |  |
| Antibiotiques par voie intraveineuse |  |  |  |  |  |
| Antibiotiques par voie orale |  |  |  |  |  |
| Sédation par voie intraveineuse |  |  |  |  |  |
| Analgésie par voie intraveineuse |  |  |  |  |  |
| Globules rouges |  |  |  |  |  |
| Plaquettes |  |  |  |  |  |
| Plasma |  |  |  |  |  |
| Traitement de suppléance de l’insuffisance rénale: hémodialyse |  |  |  |  |  |
| Traitement de suppléance de l’insuffisance rénale: dialyse péritonéale |  |  |  |  |  |

| Ressources | **Jamais** | **Rarement** | **Parfois** | **Souvent** | **Toujours** |
| --- | --- | --- | --- | --- | --- |
| Ressources financières adéquates du patient ou assurance médicale pour payer les frais des soins intensifs |  |  |  |  |  |
| Sources de référence: Manuels, ouvrages |  |  |  |  |  |
| Sources de référence: Revues médicales |  |  |  |  |  |
| Accès d’Internet fiable |  |  |  |  |  |
| Enseignement médicale continu |  |  |  |  |  |
| Partenariats/consultations avec autres institutions |  |  |  |  |  |
| Télémédicine (consultation de médecin à distance, soit par Internet, téléphone ou autre mode de communication) |  |  |  |  |  |

1. **Énumérez les quatre (4) ressources les plus utiles que vous n’avez pas à votre disposition présentement que vous pensez amélioreront grandement les soins et les résultats cliniques des patients gravement malades à votre institution.**

Ceci comprend, sans toutefois s’y limiter, des personnel de santé (p. ex. : médecins**,** infirmiers(ières), étc.), des installations (p. ex. : espace dédiée à l’USI), ou de l’équipement (p. ex. : oxygène, ventilateurs, étc.).

- 1. ________________________________________________________________________________________
  2. ________________________________________________________________________________________
  3. ________________________________________________________________________________________
  4. ________________________________________________________________________________________

1. **Dans la dernière année, avez-vous reçu des dons de médicaments ou d’équipement médicale que vous ne pouvez pas utiliser ou que vous êtes incapables d’utiliser ?**
   - Oui
   - Non

Si vous avez répondu ‘oui’ à la question précédente, veuillez élaborer :

__________________________________________________________________________________________________________________________________________________________________________________________________________________

1. **Est-ce que votre USI mène de la recherche clinique ? Veuillez sélectionner l’une des réponses suivantes :**
   - Oui
   - Non

Si vous avez répondu ‘oui’ à la question précédente, veuillez indiquer quelle sorte de recherche clinique vous avez mené :

- Rapports de cas (descriptions individuelles de patients)
- Séries de cas ou études de cohorte (examens rétrospective de dossiers)
- Comparaisons avant-après
- Essais cliniques (un traitement est comparé aux soins standards ou à un placebo)
- Autre (veuillez préciser) :______________________________________________________________

1. **D’après vous, combien d’hôpitaux en Haïti ont des unités de soins intensifs (USI) ?**

_____________________________________________________________________________________________

1. **Autres commentaires :**

_____________________________________________________________________________________________

_____________________________________________________________________________________________

_____________________________________________________________________________________________

_____________________________________________________________________________________________

_____________________________________________________________________________________________

Nous aimerions construire un réseau digital reliant des unités de soins intensifs à travers le monde. Ce réseau aiderait à planifier pendant les désastres naturels, les pandémies et les urgences, ainsi que faciliterait la recherche clinique et l’éducation.

Si vous ou vos collègues sont intéressé(e)s de faire partie d’un tel réseau, veuillez nous fournir un/des courriel(s) où nous pourrions vous rejoindre. Veuillez noter que la participation est complètement volontaire et n’est influée aucunement par l’exécution de ce sondage.

_________________________________________________________________________________________________________________

Merci beaucoup pour votre temps et votre effort.

Nous vous contacterions pour clarifier certains détails, si nécessaire.

Adapté des textes suivants :

1) Leligdowicz[, A.,](mailto:aleksandra.leligdowicz@uhn.ca) Bhagwanjee, S, Diaz, J., Xiong, W., Marshall, J.C., Fowler, R. A., and Adhikari, N.K. Development of an ICU Resource Assessment Survey for the Care of Critically Ill Patients in Resource-Limited Settings. Journal of Critical Care, 2016. and Westcott M, Martiniuk AL, Fowler RA, Adhikari NK, Dalipanda T. Critical care resources in the Solomon Islands: a cross-sectional survey. BMC International Health and Human Rights. 2012;12:1.

2) Westcott, M., Martiniuk, A.L.C., Fowler, R.A.,Adhikari, N.K.J., and Dalipanda, T.. Critical care resources in the Solomon Islands: a cross-sectional survey. BMC Int Health Hum Rights. 2012;12:1
