# Supplementary material for: Critical care capacity in Haiti: A nationwide cross-sectional survey
Source: PLoS One. 2019 Jun 13;14(6):e0218141. doi: 10.1371/journal.pone.0218141 (PMC6565360; doi:10.1371/journal.pone.0218141)
Supplement: S3 File — English version of survey instrument. (DOCX) [file pone.0218141.s008.docx]

**S3 File—Appendix 3.** English version of survey instrument.

**Haitian ICU Resource Survey for the Care of Critically Ill Patients:**

Dear Colleague,

We are sending you a survey designed to better understand critical illness in Haiti. It gathers information about you and your workplace, including resources you have to treat critically ill patients. Your responses will create a better picture of critical care in Haiti. Your participation is invaluable.

This survey will take about 15-20 minutes to complete. By participating, your consent to use your information is implied. Your responses are confidential. Please answer to the best of your ability, and feel free to consult with others if you are uncertain of any responses. If you cannot answer a particular question, leave it blank.

This study is coordinated by St. Luke’s Foundation in Port-au-Prince, Haiti and University of Maryland in Baltimore, U.S. and was reviewed by the Research Ethics Board at both institutions.

We sincerely appreciate your participation!

**1. Please tell us about yourself**

a) What is your role? (Please place X next to selection)

- - Doctor:
  - Nurse:
  - Administrator:
  - Other (Please specify): _________________________________

b) What is your highest level of education? (Please place X next to selection)

- Secondary education (i.e., high school)
- Professional training to become clinical officer
- Professional training to become nurse
- Bachelor degree (undergraduate degree in nursing or medicine)
- Medical/surgical residency training
- Subspecialty medical training or clinical fellowship
- Other: ______________________________________________

c) If you are a doctor, what is your specialty?

- Internal medicine
- Emergency Medicine
- General Surgery
- Anesthesia
- OB/Gyn
- Intensivist
- Other (please specify): __________________________________

d) If you are a physician, how many years have you been in clinical practice? (Please place X next to selection)

- 0-5 years
- 6-10 years
- 11-15 years
- 16-20 years
- >20 years

e) Do you work in more than one hospital? (Please place X next to selection)

- Yes
- No

If Yes, please refer to the hospital where you work most frequently for the remaining questions.

**2. Please tell us about your hospital**

Hospital Name: _____________________________________________________

Province/District: ____________________________________________________

City/Town: _________________________________________________________

If the hospital has a postal/zip code, please enter it: _________________________

If the hospital has a street address, please enter it: ____________________________

Phone number of your hospital (include area code and country code): ____________

a) What type of hospital is it? (Please place X next to selection)

- Academic hospital affiliated with medical school
- National referral hospital not associated with medical school
- Regional hospital
- District general hospital
- Other (Please specify): ________________________________________

b) What is the funding source? (Please place X next to selection)

- Government
- Private, not for profit (charity, missionary)
- Private, for profit (corporation)
- Military
- Other (please specify)

c) Does the hospital have an accident/emergency/casualty department? (Please place X next to selection)

- Yes
- No

d) How many beds are there in the hospital? __________________________________

(If you do not know the specific answer, please place X next to a selection below)

- <50
- 51-100
- 101-200
- 201-300
- 301-400
- >400

e) Does your hospital accept transfers from other hospitals? (Please place X next to selection)

- Never
- Rarely
- Sometimes
- Frequently
- Very Frequently

f) Does your hospital transfer patients out to a referral hospital? (Please place X next to selection)

- Never
- Rarely
- Sometimes
- Frequently
- Very Frequently

g) Which language(s) are used for patient care in your hospital? (Please place X next to all that apply)

- French
- Creole
- English
- Spanish
- Other (please specify): ________________________________________

h) How is patient care documented in your hospital? (Please place X next to all that apply)

- Paper charting
- Electronic/digital medical record
- Mixed paper and digital medical records
- Digital radiology
- Traditional film-based radiology
- Other (please specify): ________________________________________

**Please answer questions for the part of the hospital where care is most directly provided to critically ill patients.**

*Examples of critically ill patients: those with shock, severely labored breathing, or multiple traumatic injuries.

**3. Management of critically ill patients?**

1. Where in the hospital are critically ill patients managed? (Please place X next to all that apply)

- Intensive Care Unit (ICU, defined as a designated area in the hospital specifically for critically ill patients with increased nursing care)
- Post-operative recovery area
- Accident/emergency/casualty department
- Regular ward
- High acuity ward/high dependency unit (i.e. intermediate unit between ward and ICU)
- Other (Please specify): _____________________________________

b) If your hospital has an ICU, please answer the following questions.

1. How many ICU beds are available? (Please write the number of beds):

_____________________________________

1. How many nurses work in the ICU on a typical shift? Please write the number of nurses:

_____________________________________

1. Is there a doctor physically in the ICU 24 hours a day? (Please place X next to selection)

- Yes
- No

1. If no, how many hours a day does a doctor spend in the ICU?

- 1-2
- 3-5
- 6-9
- 10-12
- 13-23
- 24

1. How many of the ICU beds are occupied on an average day? (Please place X next to selection)

- 0-25%
- 26-50%
- 51-75%
- 76-100%

1. How many ICU patients can receive mechanical ventilation via an endotracheal tube at any one time? Please write number:

_____________________________________

1. How many ICU patients can receive non-invasive positive pressure ventilation via a mask (BiPAP or CPAP) at any one time? Please write number:

_____________________________________

c) How many critically ill or injured patients in total did your hospital take care of in the past week? If you have an ICU or high dependency unit, please include both patients that were admitted there, as well as any critically ill or injured patients that were cared for outside of that unit. Please write number:

_____________________________________

d) What age range of critically ill patients does your hospital care for? **(**Please place X next to all that apply)

- Neonates (<28 days of age)
- Children (28 days to 13 years of age)
- Adolescents (14-18 years of age)
- Adults (>18 years of age)

e) If you treat critically ill adolescents and adults, what is the approximate average age of ALL the critically ill adolescent and adult patients that you treated in the last 1 MONTH?

- - 14-18
  - 19-29
  - 30-50
  - 51-70
  - >70

f) If you treat critically ill children, what is approximate average age of ALL the critically ill children that you treated in the last 1 MONTH?

- Neonate (< 28 days)
- 1 month-11 months
- 1-5 years
- 6-10 years
- 11-14 years

g) Who provides care to critically ill patients at the bedside EVERY DAY?

(Please place X next to all that apply, even if only for a portion of the day)

- Doctor (if present, what are the doctors’ specialties? Please place X next to all that apply)
  - Anesthesia
  - Internal Medicine
  - Pulmonology
  - Surgery
  - Intensive Care
  - Pediatrics
  - Generalist/General practitioner
  - Other specialty (please specify): ______________________________
- Doctor’s assistant (such as clinical officer)
- Nurse
- Respiratory therapist
- Pharmacist
- Patient’s family or friends
- Other personnel (Please specify) : _________________________________

h) Of the doctors that care for critically ill or injured patients, do they have the following training?

- Formal critical care training (such as fellowship)
- Informal critical care training (such as experience in a foreign country ICU)
- Have taken basic critical care course (such as ATLS, ACLS, PALS, EDICS, BASIC, etc)
- Other (please specify): ___________________________________________

i) Of the nurses that care for critically ill or injured patients, do they have the following training?

- Formal critical care training
- Informal critical care training (such as experience in a foreign country ICU)
- Have taken basic critical care course (such as ATLS, ACLS, PALS, EDICS, BASIC, etc)

Other (please specify): _____________________________________________

j) How commonly do you treat patients with critical illness caused by each condition below? For each condition, please check Never, Rarely, Sometimes, Often, or Always

|  | **Never** | **Rarely** | **Sometimes** | **Often** | **Always** |
| --- | --- | --- | --- | --- | --- |
| Motor Vehicle Collisions |  |  |  |  |  |
| Maternal complications of pregnancy and childbirth |  |  |  |  |  |
| Burns |  |  |  |  |  |
| Toxins (for example, poisoning, snake bites) |  |  |  |  |  |
| Interpersonal Violence (for example, assaults, homicide) |  |  |  |  |  |
| Self Inflicted injuries (for example, suicide attempts) |  |  |  |  |  |
| Other traumatic accidents (for example, falling off roof, drowning) |  |  |  |  |  |
| Illness in newborn children |  |  |  |  |  |
| Tuberculosis |  |  |  |  |  |
| Malaria |  |  |  |  |  |
| Tetanus |  |  |  |  |  |
| Motor Vehicle Collisions |  |  |  |  |  |
| HIV related illness |  |  |  |  |  |
| Congestive heart failure |  |  |  |  |  |
| Ischemic heart disease |  |  |  |  |  |
| Congenital heart disease |  |  |  |  |  |
| Rheumatic heart disease |  |  |  |  |  |
| Nutritional disease |  |  |  |  |  |
| Cancer |  |  |  |  |  |
| Chronic lung disease (for example, asthma, COPD) |  |  |  |  |  |
| Acute disease of respiratory system (for example, pneumonia, ARDS) |  |  |  |  |  |
| Ischemic Stroke |  |  |  |  |  |
| Hemorrhagic stroke |  |  |  |  |  |
| Conditions leading to elective operations |  |  |  |  |  |
| Conditions leading to emergent operations |  |  |  |  |  |
| Complications of operations |  |  |  |  |  |
| Sepsis |  |  |  |  |  |
| Renal Failure |  |  |  |  |  |
| Diabetes mellitus |  |  |  |  |  |
| Gastrointestinal bleeding |  |  |  |  |  |
| Cholera |  |  |  |  |  |
| Shock |  |  |  |  |  |
| Mental illness |  |  |  |  |  |
| Other 1 (please specify): |  |  |  |  |  |
| Other 2 (please specify): |  |  |  |  |  |

k) Of the conditions listed in the table above, please rank and list the top 5 causes of death of critically ill patients at your hospital:

1) _____________________________________________

2) _____________________________________________

3) _____________________________________________

4) _____________________________________________

5) _____________________________________________

l) What resources are available in your hospital to care for critically ill patients? For each condition, please check Never, Rarely, Sometimes, Often, or Always.

| Personnel | **Never** | **Rarely** | **Sometimes** | **Often** | **Always** |
| --- | --- | --- | --- | --- | --- |
| More nurses per patient than on a general ward: |  |  |  |  |  |
| Pediatrician |  |  |  |  |  |
| Obstetrician |  |  |  |  |  |
| Surgeon |  |  |  |  |  |
| Respiratory therapist |  |  |  |  |  |
| Pharmacist |  |  |  |  |  |
| Subspecialist Surgeon (ie, neurosurgeon, ENT, urologist) |  |  |  |  |  |
| Intensivist |  |  |  |  |  |
| Microbiologist |  |  |  |  |  |

| Equipment | **Never** | **Rarely** | **Sometimes** | **Often** | **Always** |
| --- | --- | --- | --- | --- | --- |
| Hospital beds/stretchers |  |  |  |  |  |
| Operating room/theater |  |  |  |  |  |
| Reliable electricity |  |  |  |  |  |
| Electrical generator back up |  |  |  |  |  |
| Reliable and safe water supply |  |  |  |  |  |
| Soap |  |  |  |  |  |
| Gloves |  |  |  |  |  |
| Oxygen by pipe |  |  |  |  |  |
| Oxygen by cylinder |  |  |  |  |  |
| Oxygen by concentrator |  |  |  |  |  |
| Pulse oximetry |  |  |  |  |  |
| Non-invasive ventilation (BiPAP, CPAP) |  |  |  |  |  |
| Invasive mechanical ventilation |  |  |  |  |  |
| Nasogastric tubes |  |  |  |  |  |
| Chest tubes |  |  |  |  |  |
| Arterial blood gas analysis |  |  |  |  |  |
| EKG machines |  |  |  |  |  |
| Automated blood pressure monitor |  |  |  |  |  |
| Automated Vital Sign Monitor Screens |  |  |  |  |  |
| Defibrillators |  |  |  |  |  |
| X-Ray machine (not portable) |  |  |  |  |  |
| Portable X-Ray machine |  |  |  |  |  |
| Portable (bedside) ultrasound machine |  |  |  |  |  |
| CT Scan |  |  |  |  |  |
| MRI Scan |  |  |  |  |  |
| Negative pressure isolation rooms |  |  |  |  |  |
| Microbiology laboratory |  |  |  |  |  |
| Arterial catheters |  |  |  |  |  |
| Central venous catheter |  |  |  |  |  |
| Peripheral intravenous catheters |  |  |  |  |  |

| Therapy | **Never** | **Rarely** | **Sometimes** | **Often** | **Always** |
| --- | --- | --- | --- | --- | --- |
| Intravenous crystalloid fluids |  |  |  |  |  |
| Intravenous inotropes or vasopressors |  |  |  |  |  |
| Intravenous antibiotics |  |  |  |  |  |
| Oral antibiotics |  |  |  |  |  |
| Intravenous sedation |  |  |  |  |  |
| Intravenous analgesia |  |  |  |  |  |
| Packed red blood cells |  |  |  |  |  |
| Platelets |  |  |  |  |  |
| Plasma |  |  |  |  |  |
| Renal Replacement therapy: Hemodialysis |  |  |  |  |  |
| Renal Replacement therapy: Peritoneal Dialysis |  |  |  |  |  |

| Resources | **Never** | **Rarely** | **Sometimes** | **Often** | **Always** |
| --- | --- | --- | --- | --- | --- |
| Adequate patient finances or health insurance to pay for intensive care |  |  |  |  |  |
| Reference information: Textbooks |  |  |  |  |  |
| Reference information: Medical Journals |  |  |  |  |  |
| Reliable internet access |  |  |  |  |  |
| Continuing health education |  |  |  |  |  |
| Partnerships/consultation with other institutions |  |  |  |  |  |
| Telemedicine (ie, remote consultants available over internet, phone, or other device) |  |  |  |  |  |

**4) Please list the four (4) most useful resources that you currently do not have that you think would greatly improve the care and outcome of critically ill patients in your hospital?** Examples include, but are not limited to, personnel (doctors, nurses, etc.), facilities (dedicated ICU space, etc.), or equipment (oxygen, tubing, ventilators, etc.).

1. ___________________________________________

2. ___________________________________________

3. ___________________________________________

4. ___________________________________________

**5) In the past year, have you received donations of medications or medical equipment that you cannot or are unable to use?**

- Yes
- No

If yes to the above question, please explain:

____________________________________________________________________________________________________________________________________________________________

**6) Does your ICU conduct clinical research? (Please place X next to selection)**

- Yes
- No

If yes, what kind of clinical research have you conducted?

- Case reports (individual patient descriptions)
- Case series or cohort studies (chart reviews)
- Before-after comparisons
- Clinical Trials (treatment is compared to standard care or a placebo)
- Other (please specify): ___________________________________________

**7) How many hospitals in Haiti do you think have intensive care units (ICUs)?**

___________________________________________

**8) Any other comments:**

___________________________________________

___________________________________________

___________________________________________

___________________________________________

We would like to build an electronic network linking ICUs around the world. This network would help to plan during disasters, pandemic and emergencies; facilitate clinical research; and facilitate education.

If you and or your colleagues would be interested in being a part of such a network, please provide an email address or addresses where you can be reached. Note that participation is completely voluntary and has no bearing on completion of this survey.

______________________________________________________________________

Thank you very much for your time and effort.

We may contact you again to clarify details.

Modified from:

1) Leligdowicz[, A.,](mailto:aleksandra.leligdowicz@uhn.ca) Bhagwanjee, S, Diaz, J., Xiong, W., Marshall, J.C., Fowler, R. A., and Adhikari, N.K. Development of an ICU Resource Assessment Survey for the Care of Critically Ill Patients in Resource-Limited Settings. Journal of Critical Care, 2016. and Westcott M, Martiniuk AL, Fowler RA, Adhikari NK, Dalipanda T. Critical care resources in the Solomon Islands: a cross-sectional survey. BMC International Health and Human Rights. 2012;12:1.

2) Westcott, M., Martiniuk, A.L.C., Fowler, R.A.,Adhikari, N.K.J., and Dalipanda, T.. Critical care resources in the Solomon Islands: a cross-sectional survey. BMC Int Health Hum Rights. 2012;12:1
